# Vestibular modulation of muscle sympathetic nerve activity during sinusoidal linear acceleration in supine humans

**DOI:** 10.3389/fnins.2014.00316

**Published:** 2014-10-09

**Authors:** Elie Hammam, Philip S. Bolton, Kenny Kwok, Vaughan G. Macefield

**Affiliations:** ^1^School of Medicine, University of Western SydneySydney, NSW, Australia; ^2^School of Biomedical Sciences and Pharmacy, University of NewcastleNewcastle, NSW, Australia; ^3^Hunter Medical Research InstituteNewcastle, NSW, Australia; ^4^Institute for Infrastructure Engineering, University of Western SydneySydney, NSW, Australia; ^5^Neuroscience Research AustraliaSydney, NSW, Australia

**Keywords:** MSNA, sympathetic, utricle, saccule, vestibulosympathetic reflexes

## Abstract

The utricle and saccular components of the vestibular apparatus preferentially detect linear displacements of the head in the horizontal and vertical planes, respectively. We previously showed that sinusoidal linear acceleration in the horizontal plane of seated humans causes a pronounced modulation of muscle sympathetic nerve activity (MSNA), supporting a significant role for the utricular component of the otolithic organs in the control of blood pressure. Here we tested the hypothesis that the saccule can also play a role in blood pressure regulation by modulating lower limb MSNA. Oligounitary MSNA was recorded via tungsten microelectrodes inserted into the common peroneal nerve in 12 subjects, laying supine on a motorized platform with the head aligned with the longitudinal axis of the body. Slow sinusoidal linear accelerations-decelerations (peak acceleration ±4 mG) were applied in the rostrocaudal axis (which predominantly stimulates the saccule) and in the mediolateral axis (which also engages the utricle) at 0.08 Hz. The modulation of MSNA in the rostrocaudal axis (29.4 ± 3.4%) was similar to that in the mediolateral axis (32.0 ± 3.9%), and comparable to that obtained by stimulation of the utricle alone in seated subjects with the head vertical. We conclude that both the saccular and utricular components of the otolithic organs play a role in the control of arterial pressure during postural challenges.

## Introduction

The roles of the vestibular system in eye coordination (vestibulo-occular reflexes) and postural stability (vestibulospinal reflexes) have been extensively studied. An emergent role of the vestibular system has been in its interaction with the sympathetic nervous system (vestibulosympathetic reflexes) to modulate, for example, blood flow to the limbs (for review see Yates et al., 2014). The vestibular apparatus is comprised of two sets of organs, the semicircular canals—sensitive to angular acceleration about three orthogonal axes—and the otoliths. The latter consist of the utricle and saccule, which are preferentially sensitive to linear acceleration in the horizontal and vertical planes, respectively. The origin of the vestibular modulation of sympathetic outflow remains equivocal, though it is generally accepted that the otoliths provide the most important source of sensory input to the vestibular system (Yates et al., 1993; Ray et al., 1998; Cohen et al., 2012; Holstein et al., 2012).

Over the last decade, we have been using galvanic vestibular stimulation (GVS) in awake human subjects as a means of selectively activating the vestibular primary afferents (Minor and Goldberg, 1991; Fitzpatrick and Day, 2004) without altering other inputs to the cardiovascular control system. Sinusoidal GVS at frequencies between 0.2 and 2.0 Hz revealed partial phase locking of sympathetic outflow to both muscle (Grewal et al., 2009) and skin (James et al., 2010). However, GVS modulates afferent activity from the entire vestibular apparatus, and whilst there is considerable evidence supporting the otoliths being the source of the vestibulosympathetic responses (Yates et al., 1993), even during sinusoidal GVS (Cohen et al., 2012; Holstein et al., 2012), we do not know whether the modulation is of utricular and/or saccular origin. To further investigate the afferent source of the vestibulosympathetic reflexes, we recently employed physiological stimulation to target the utricle selectively: applying slow sinusoidal linear accelerations (0.08 Hz) in the horizontal plane to subjects seated (head vertical) on a motorized platform showed that sympathetic activity to both muscle (Hammam et al., 2013, 2014) and skin (Grewal et al., 2012) was sinusoidally modulated in a manner similar to that we had seen with sinusoidal GVS. In the current study we sought to investigate whether physiological activation of the saccule—which normally is sensitive to acceleration in the vertical (gravitational) plane—affects sympathetic outflow to muscle. Of course, the otoliths are not flat surfaces but curved structures, both in the guinea pig (Curthoys et al., 1999) and human (Curthoys et al., 2009), such that some afferents within the utricular component will respond to acceleration in the vertical plane, and some within the saccular component will be sensitive to motion in the horizontal plane. However, given that the majority of the afferents in the utricular macula will be aligned in the vertical plane when the body and head are supine, it is reasonable to assume that sinusoidal linear acceleration of the supine body in the rostrocaudal axis will preferentially activate afferents in the saccular macula. Accordingly, we tested the hypothesis that preferential activation of saccular afferents in the supine position evokes greater modulation of muscle sympathetic nerve activity (MSNA) to the lower limbs than does preferential activation of utricular afferents, induced by acceleration in the horizontal plane when the body and head are vertical (Hammam et al., 2013, 2014).

Given that acceleration in the rostro-caudal direction simulates hemodynamic shifts during vertical displacement (e.g., traveling in an elevator) in the upright position, it is likely that the saccular contribution to blood pressure regulation is more important along this axis than in the mediolateral axis, which simulates fluid shifts from side to side. Importantly, by conducting these studies in supine subjects with the head aligned in the longitudinal axis we avoided superimposition of the imposed accelerations on the much stronger gravitational field, which remains constant when accelerations are applied to the supine body in the horizontal plane.

## Methods

The study was conducted with the approval of the Human Research Ethics Committee, University of Western Sydney, and satisfied the Declaration of Helsinki. Experiments were performed on 12 subjects (8 male, 4 female; 19 to 54 years), each of whom provided written informed consent. Prior to the experiments, participants were asked to avoid any caffeinated beverages for 3 h before the experiment. None of the participants smoked. Subjects laid supine on a comfortable bed, neck aligned with the spine. The head was stabilized with a Velcro strap and padding to avoid rotational movements. A blindfold, earplugs and earmuffs were applied to prevent any visual or auditory cues. The bed was surmounted on a motorized platform driven by two linear motors that had a maximal excursion of ±20 cm in the rostrocaudal (X) and mediolateral (Y) directions. Accelerations were measured using two high-sensitivity accelerometers, with a threshold of <10 μG (QA650, Honeywell, USA), fixed to the platform—one orientated in the X-axis, the other in the Y. Sinusoidal movements of the platform, with a peak to peak excursion of 15 cm, were delivered separately in the X or Y directions at 0.08 Hz and 4 mG for 100 cycles. Because of the arrangement of the hair cells within the otoliths, accelerations in the X-axis (rostrocaudal) cause displacement of most hair cells within the saccule, whilst movements along the Y-axis (mediolateral) should cause displacements of most of the hair cells within the utricle, yet relatively few cells in the saccule. Subjects were asked to provide their perceptions at the conclusion of each stimulation sequence.

MSNA was recorded from muscle fascicles of the left common peroneal nerve via a tungsten microelectrode (FHC, Bowdoinham, ME, USA) inserted percutaneously at the fibular head; an uninsulated reference microelectrode was inserted subdermally ~1 cm away. Intraneural stimulation (0.01–1.0 mA, 1 Hz, 0.2 ms pulses), delivered to the microelectrode via an isolated stimulator (Stimulus Isolator, ADInstruments, Sydney, Australia) was used to guide the microelectrode tip into a motor fascicle of the nerve. Neural activity was amplified (gain 20,000, bandpass 0.3–5.0 kHz; NeuroAmp EX, ADInstruments, Sydney, Australia) and the microelectrode tip manually advanced toward spontaneous bursts of oligounitary MSNA. MSNA was defined as such if it occurred with a clear cardiac rhythmicity, did not respond to unexpected arousal stimuli, and increased its activity during a maximal inspiratory apnoea. Neural activity was stored on a computer (10 kHz sampling) using a computer-based data acquisition and analysis system (PowerLab 16SP hardware and LabChart 7 software; ADInstruments, Sydney, Australia). Other measurements included ECG (0.3–1.0 kHz), recorded with Ag–AgCl surface electrodes on the chest and sampled at 2 kHz, respiration (DC-100 Hz), recorded via a piezoelectric transducer (Pneumotrace, UFI, Morro Bay CA, USA) wrapped around the chest, and non-invasive continuous blood pressure recorded from a finger (Finometer, Finapres Medical Systems, The Netherlands). Baseline activity was recorded for 5 min followed by acceleration of the platform, separately, in the X or Y directions at 0.08 Hz for 100 cycles (~21 min each).

MSNA was displayed as the raw neurogram as well as the RMS-processed (root mean square, moving average over 200 ms) MSNA signal. However, as described previously (Bent et al., 2006), the primary analysis was conducted on the raw, negative-going, sympathetic spikes. Negative-going spikes in the neurogram (with a half-width of 0.2–0.5 ms), positive-going spikes in the ECG and the positive peaks of the accelerometer signals were detected using window discriminator software (Spike Histogram for Macintosh v2.2, ADInstruments, Sydney, Australia); this same software was used to construct cross-correlation and auto-correlation histograms (autocorrelograms). Cross-correlation histograms (50 ms bins) were constructed between MSNA and the positive peaks of the accelerometer signals in the X or Y directions (100 cycles at 0.08 Hz; ~21 min for each axis). The histogram data was exported to a statistical and graphical analysis program (Prism 5.0 for Macintosh, GraphPad Software, USA) to fit the data to a smoothed polynomial. Lower-order polynomials were used to fit curves to the cardiac cross-correlation histograms while higher-order polynomials were required to fit curves to the slower vestibular cross-correlation histograms. The polynomials were adjusted to create a smooth graph of the cross-correlograms. The purpose of smoothing cross-correlation histograms between sympathetic nerve activity and platform motion is to eliminate any cardiac related peaks. This enables us to further examine the nerve activity more accurately with respect to the sinusoidal platform motion. The modulation of MSNA was quantified by measuring the difference in the number of spikes on the smoothed curve at the peak of the modulation and at the trough. This figure was then expressed as a percentage by employing the following formula: modulation index (%) = [(peak—trough)/peak] × 100. Comparisons were then made between the mean modulation values of each type of platform motion across all individuals. Paired *t*-tests were used to compare differences in vestibular and cardiac modulation indices in the X and Y-axes (Prism 6 for Mac, GraphPad Software Inc., USA). The level of statistical significance was set at *p* < 0.05.

## Results

Stable oligounitary recordings of MSNA were obtained in all 12 supine subjects during slow sinusoidal linear acceleration (±4 mG, 0.08 Hz) in the rostrocaudal direction (X-axis), i.e., along the longitudinal axis of the supine body, but (due to technical problems) in only 10 in the mediolateral direction (Y-axis). No subjects could detect motion in the X-axis, but all reported motion when the sinusoidal stimuli were delivered side-to side; however, the majority (8/10) could not determine the direction of movements. No subjects reported any discomfort or nausea.

**Figure 1:**
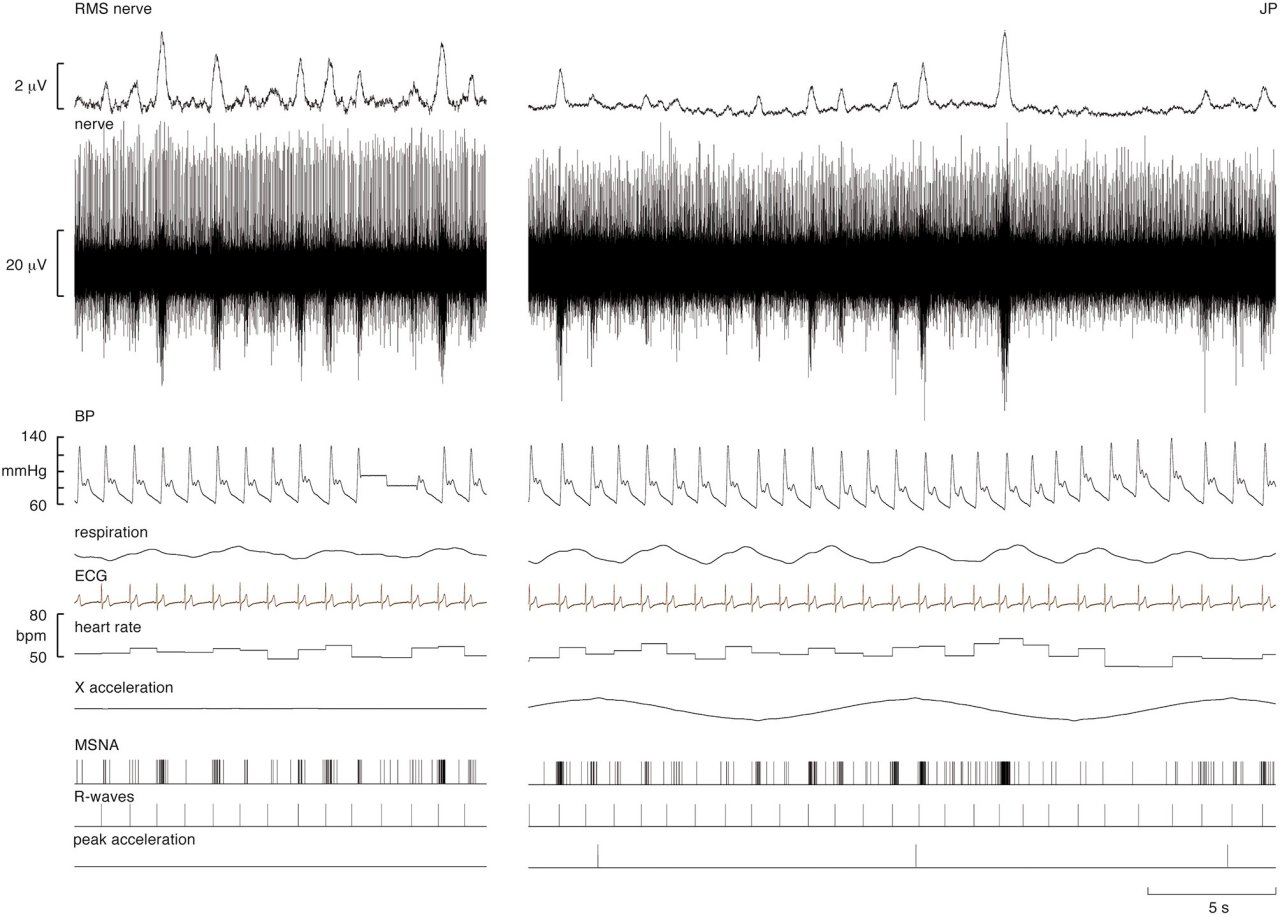
**Experimental records from a supine male subject before and during sinusoidal linear acceleration in the X-direction (i.e., in the longitudinal axis of the body)**. The nerve recording includes a spontaneously active muscle spindle (positive-going spikes) and spontaneous bursts of muscle sympathetic nerve activity (MSNA), reflected in the RMS-processed signal. Negative-going sympathetic spikes are shown discriminated below (MSNA); these were used to generate the cross-correlation histograms between the vestibular (peak acceleration) or cardiac (R-waves) signals. The acceleration (4 mG) caused no overt changes in heart rate, blood pressure, or respiration. The slow baseline deflections in the RMS nerve trace reflect cable movements from the headstage; these had no effect on the discriminated sympathetic spikes.

Experimental records from one subject are shown in Figure 1. During the period of sinusoidal linear acceleration-deceleration there were no overt changes in heart rate, blood pressure or respiration. It can be seen that the microelectrode picked up positive-going spikes from a tonically-active muscle spindle afferent as well as negative-going spikes occurring as bursts of MSNA. The negative-going spikes have been discriminated and represented as standard pulses, shown in the lower traces; these were used to generate cross-correlation histograms between MSNA and the positive peaks of the acceleration signal, and between MSNA and the R-waves of the ECG, also shown in the lower traces.

**Figure 2:**
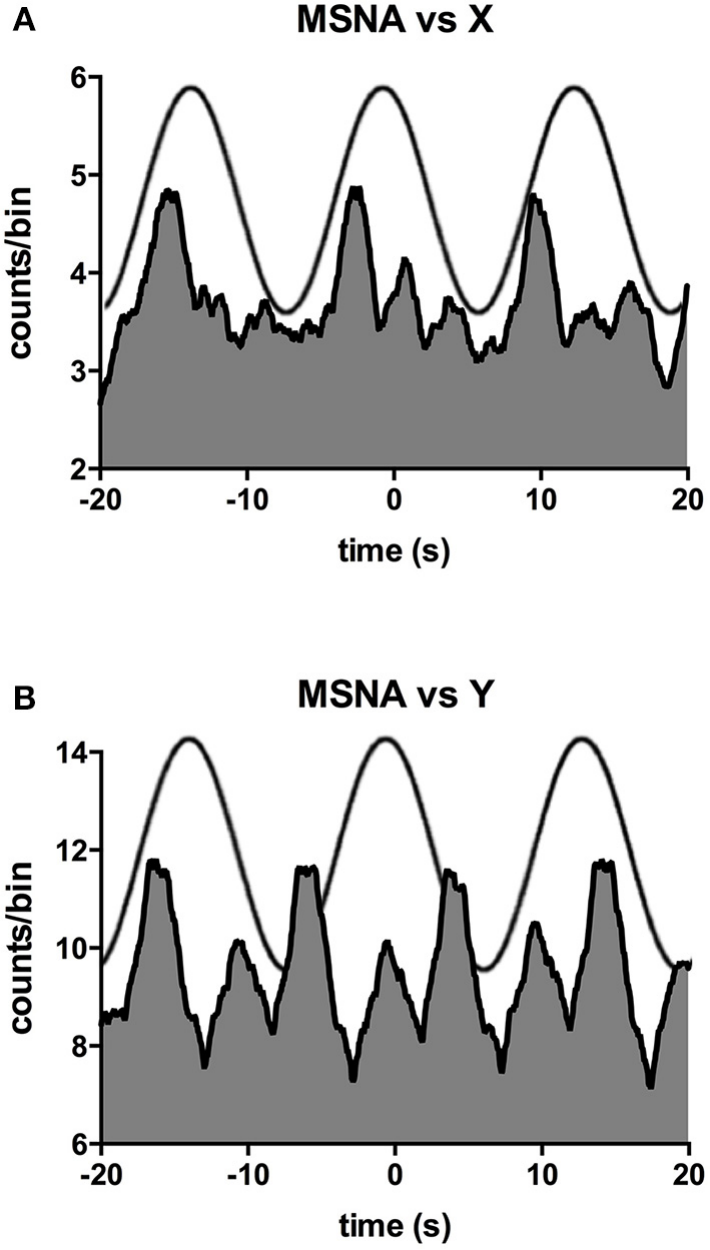
**Cross-correlation histograms between MSNA and acceleration in the rostrocaudal (X) axis (A) and mediolateral (Y) axis (B) in a male subject**. The histograms have been fitted with a smoothed polynomial. The superimposed sinusoid represents the acceleration of the platform in the rostrocaudal **(A)** or mediolateral **(B)** axes.

**Figure 3:**
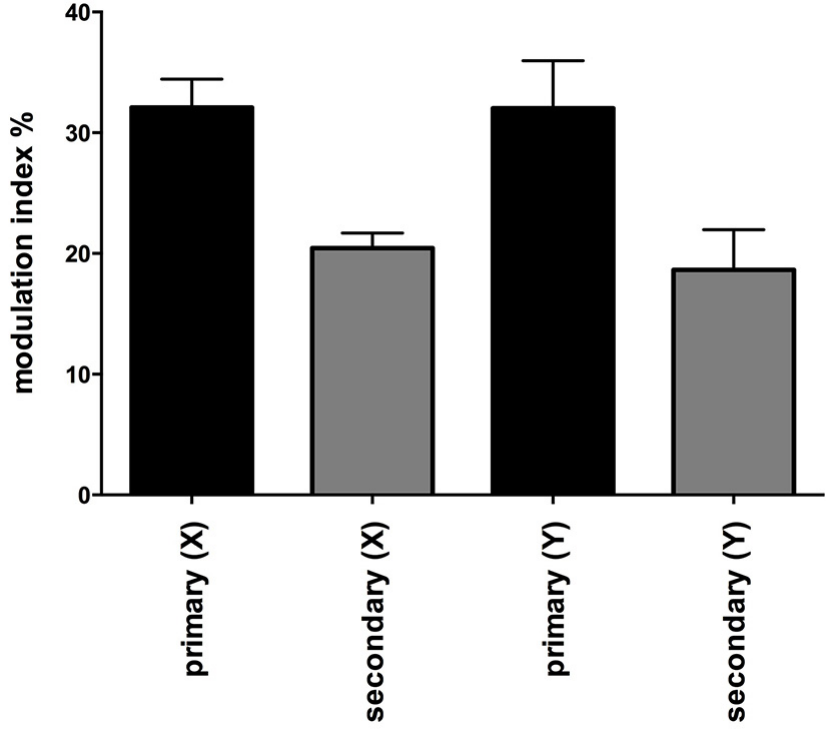
**Mean modulation indices of primary and secondary peaks as a function of direction of sinusoidal motion**. Modulation indices were significantly higher for the primary than the secondary peaks, but there was no difference in the magnitude of either component between axes. Secondary peaks were not expressed in all subjects. Mean ± SE: *n* = 12 for the primary peak, *n* = 7 for the secondary peak in the X axis and 5 in the Y axis.

Figure 2 shows one subject's cross-correlation analysis between MSNA and displacements in the rostrocaudal (A) and the mediolateral (B) axes. It is clear that there is a single large peak of modulation of MSNA for each cycle of stimulation (the primary peak) and a smaller (secondary) peak. The secondary peaks were more variable, found in 7 of the 11 subjects during acceleration in the X-axis and in 5 of 9 subjects in the Y-axis. Across subjects the vestibular modulation of MSNA was significant in both axes (*p* < 0.0001). Mean modulation indices of the primary and secondary peaks are shown graphically in Figure 3. Whilst the secondary peaks were significantly smaller than the primary peaks in both axes (*p* < 0.0001), there was no difference in the magnitudes of either the primary or secondary peaks in each axis of motion. Moreover, there were no significant effects of the direction of motion on the latency of either the primary and secondary peaks.

**Figure 4:**
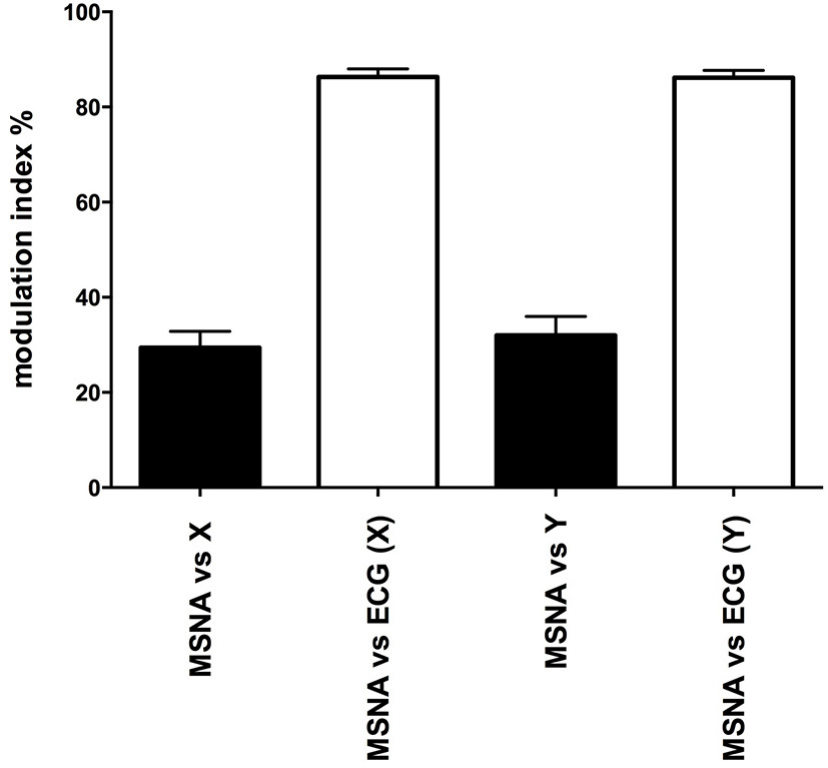
**Mean ± SE modulation indices for the cardiac entrainment of MSNA (white) and vestibular modulation (black)**. Mean data obtained from 12 (X-axis) and 10 (Y-axis) subjects.

Mean data for the magnitudes of vestibular (primary peak) and cardiac modulation are presented in Table 1. As expected, the magnitude of the vestibular modulation of MSNA was significantly smaller than that of the cardiac modulation (*p* < 0.0001). This was true regardless of whether the acceleration was applied in the rostrocaudal (29.4 ± 3.4%) or mediolateral (32.0 ± 3.9%) axes (Figure 4).

**Table 1:** **Vestibular and cardiac modulation of MSNA during sinusoidal acceleration**.

**MSNA**	**X axis**	**Y axis**
Vestibular modulation (%)	29.4 ± 3.4	32.0 ± 3.9
Cardiac modulation (%)	86.3 ± 1.7	86.2 ± 1.6
*n*	12	10

*Modulation Index = [(peak-trough)/peak] × 100, calculated from the smoothed cross-correlation histograms (primary peak) between MSNA and the vestibular or cardiac inputs. Mean ± SEM data in the X-axis. There was no significant difference between the respective modulation indices. N, number of subjects*.

## Discussion

This study has demonstrated that low-frequency, low-amplitude, sinusoidal linear accelerations of the supine body—which primarily activates the saccular hair cells—causes a marked modulation of MSNA. However, the magnitude of this modulation (29% in the X-axis) was no different to that produced by selective stimulation of the utricle in seated subjects with head vertical (32% in the anteroposterior axis, 29% in the mediolateral axis; Hammam et al., 2013). In addition, the vestibular modulation of MSNA was not significantly different when the stimuli were applied in the mediolateral axis of supine subjects (32%), which engages the utricular hair cells and only minimally engages the saccular hair cells. This argues against a *dominant* role of the saccule in modulating MSNA in humans, a hypothesis we predicted in our earlier study (Hammam et al., 2013); rather, the saccule and utricle would both appear to contribute, which makes sense in terms of the cardiovascular adjustments required during changes in posture and hence head position with respect to the gravitational vector.

In the erect posture the saccular macula is aligned in the sagital plane and is slightly concave medially; most saccular hair cells are activated maximally by vertical displacements, although some are maximally activated by anteroposterior displacements (Fitzpatrick and Day, 2004). In contrast, the utricular macula is inclined backwards 30° from the horizontal and its superior surface is concave, with most of its hair cells being maximally activated by mediolateral displacements and relatively few being maximally activated by displacements in the sagital (vertical) plane (Fitzpatrick and Day, 2004). Moreover, it has been shown that whereas the entire saccule is fixed to the temporal bone, only the rostral part of the utricle is rigidly attached to the bone—the majority of the utricular macula is only loosely supported (Curthoys et al., 2009). Accordingly, it has been suggested that afferents in the more caudal part of the utricle may respond to “deflections of the entire macula as well as by local shearing due to shift of the otoconial membrane” (Curthoys et al., 2009). While we could have undertaken the current study during vertical displacements of the erect body (seated or standing), such as occurs when traveling in an elevator, not withstanding the methodological problems inherent in this approach this would incur gravitational loads on the hydrostatic column and hence influence the baroreceptors. Given that the baroreceptors provide the dominant source of modulation of MSNA, any increases in baroreceptor input would thereby affect our ability to measure the saccular modulation of MSNA.

Accordingly, to reduce such gravitationally-induced fluid shifts we delivered sinusoidal linear acceleration in the rostrocaudal axis in supine subjects. Based on evidence from non-human primate studies of vestibular afferent activation (Fernandez and Goldberg, 1976), in this position the saccular macula remains aligned in the sagital plane, but the 90° posterior rotation of the head from the vertical position aligns the saccular macula such that most of its hair cells will be maximally activated in the horizontal rather than the vertical (gravitational) plane. By contrast, the utricular macula will be tilted ~30° posterior to the coronal plane and, although the direction of maximal activation for most of its hair cells remains in the mediolateral axis, they are now in the coronal rather than horizontal plane. The remaining few utricular hair cells that are maximally activated by displacement in the vertical (sagital) plane will now be activated in the horizontal plane. Furthermore, the polarity of maximal activation of hair cells on one side of the macular striola is opposite to that on the other, such that movement in opposite directions activates hairs cells on one side of the striola while inhibiting those on the other. Hence, during sinusoidal accelerations in the horizontal plane both directions of displacements (rostrocaudal or mediolateral) will be detected. In this way we believe we have predominately activated the saccular hair cells when displacing our supine subjects in the rostro-caudal axis, with an additional contribution from utriclar hair cells during mediolateral excursions. Consistent with this was the finding in our study that many subjects exhibited two peaks of MSNA, presumably arising from the acceleration (and hence activation) imposed on the hair cells on opposite sides of the striola during the changes in direction of the sinusoidal acceleration. Importantly, the low frequency nature of the stimulus employed largely reduces extra-vestibular afferent firing, allowing us to examine the otolithic contribution to the vestibulosympathetic reflex essentially in isolation.

We should acknowledge that previous studies have employed linear accelerations to demonstrate the contribution of the otolithic organs to cardiovascular control. Yates et al. (1999) showed increases in blood pressure and heart rate during linear accelerations—though these were at much higher levels (200 mG) than those used in the current study (4 mG)—and these were absent in patients with idiopathic profound bilateral reduction in vestibular function. Similar results were found by Jauregui-Renaud et al. (2006); control subjects showed a sustained increase in heart rate, and a transient increase in respiration, when exposed to brisk linear accelerations (260 mG), while patients with chronic bilateral vestibular dysfunction did not. These studies, amongst others (Yates et al., 1999; Radtke et al., 2000, 2003; Jauregui-Renaud et al., 2003, 2005) strongly support the contribution of the otolithic organs to cardiovascular adjustments. Furthermore, Cui et al. (2001) recorded MSNA during five cycles of sinusoidal linear accelerations of varying amplitude (100, 150, 200 mG) and found that these higher magnitudes of accelerations actually *decreased* total MSNA, in both the anteroposterior and mediolateral directions (Cui et al., 1999, 2001).

By employing linear accelerations at much smaller magnitudes of acceleration (4 mG), which is below perceptual threshold (Hammam et al., 2014), we recently demonstrated that selective activation of the vestibular utricle—produced by delivering linear accelerations in the horizontal plane to seated subjects, causes a marked modulation of MSNA (Hammam et al., 2013, 2014) as well as SSNA (Grewal et al., 2012). As noted above, the magnitudes of modulation of MSNA are similar to those seen in the current study, and similar to those observed during electrical stimulation of the vestibular nerves with sinusoidal GVS at 0.08 Hz (Hammam et al., 2011). This affirms the observations by Cohen et al. (2012; Holstein et al., 2012)—that the vestibulosympathetic reflex evoked by galvanic stimulation originates in the otolithic organs, not the semicircular canals. It is also worth reiterating that whilst acceleration along the rostrocaudal axis predominantly stimulates the saccule (containing approximately half the number of hair cells than that of the utricle), whereas acceleration along the mediolateral axis excites hairs cells in the utricle, the magnitude of modulation did not differ, leading us to the conclusion that the vestibulosympathetic reflex is not a sole function of one set of otolithic end-organs, *but a result of hair cell firing from one or both*. Furthermore, whilst the modulation indices in both axes showed similar distributions across subjects, many individuals exhibited larger changes in sympathetic modulation during some stimulus conditions than others. We do not know why this is the case, but it has been suggested by Yates et al. (1999) that “shaping” and “tuning” of hair cell responses based on experiences may impact on individual effects; this hypothesis remains to be tested.

As expected, cardiac modulation of MSNA was much higher than the vestibular modulation; this is simply due to the strong coupling of MSNA to the cardiac rhythm via the baroreflex (James and Macefield, 2010). Nevertheless, as noted previously, the vestibular modulation of MSNA does appear to be independent of the cardiac modulation, but competes with inputs from the arterial baroreceptors when the frequencies of vestibular stimulation are closer to that of the cardiac rhythm (James and Macefield, 2010); in the current study, the sinusoidal stimulus (0.08 Hz) was far removed from the cardiac frequency (~1 Hz). In addition, the current study has demonstrated that saccular inputs, as well as utricular inputs, induce modulation of muscle vasoconstrictor drive, which presumably acts through projections from the vestibular hair cells to the vestibular nuclei onto the rostral ventrolateral medulla (RVLM) (Holstein et al., 2011), the primary output nucleus for muscle vasoconstrictor neurones (Dampney et al., 2003a,b), which has been shown to receive excitatory inputs from the vestibular apparatus, primarily from the otoliths (Yates et al., 1991, 1993).

## Limitations

Our aim in this study was to examine the magnitude of MSNA modulation during preferential activation of saccular hair cells. In the absence of a safe non-invasive method to selectively stimulate hair cells in the human saccule, we employed an indirect strategy based on the functional anatomy of the otoliths (by displacing subjects horizontally when supine) to predominately stimulate the hair cells in the saccule or, as described previously, in the utricle (Grewal et al., 2012; Hammam et al., 2013, 2014). The challenge remains that we cannot conclusively eliminate the possibility of having activated other mechanoreceptors sensitive to acceleration, such as the graviceptors (Mittelstaedt, 1992). However, as noted above, the magnitude of vestibular modulation induced by sinusoidal electrical stimulation (GVS) of the vestibular nerves at the same frequency (Hammam et al., 2011, 2012) is very similar to that produced by physiological stimulation of the utricle (Hammam et al., 2013) or saccule, hence supporting our interpretation that we are stimulating the otoliths essentially selectively. We would expect that vestibular modulation of MSNA would be absent in patients with bilateral damage to the hair cells, though acknowledge that non-vestibular inputs may also contribute. Indeed, it has been shown that neurones in the vestibular nuclei can still respond to tilt in labrynthectomized cats (Yates et al., 2000), and we recently showed that sinusoidal stimulation of the neck muscles can also induce potent modulation of MSNA (Bolton et al., 2014).

## Conclusions

We have shown that physiological stimulation of the otoliths, produced by sinusoidal linear acceleration in supine subjects causes a marked modulation of MSNA. We have argued that stimuli applied in the rostro-caudal axis preferentially excites afferents in the saccule, and shown that the modulation of MSNA is of comparable magnitude to that produced by selective stimulation of the utricle, produced by delivering sinusoidal linear acceleration to seated subjects. Evidently, both sets of otolithic organs contribute to the control of muscle vasoconstrictor drive, and hence both are involved in the regulation of blood pressure.

### Conflict of interest statement

The authors declare that the research was conducted in the absence of any commercial or financial relationships that could be construed as a potential conflict of interest.
